# Health effectiveness and cost-effectiveness of telehealthcare for heart failure: study protocol for a randomized controlled trial

**DOI:** 10.1186/s13063-016-1722-5

**Published:** 2016-12-12

**Authors:** Simon Lebech Cichosz, Lars Holger Ehlers, Ole Hejlesen

**Affiliations:** 1 Department of Health Science and Technology, Aalborg University, Fredrik Bajers Vej 7D2, DK-9220 Aalborg, Denmark; 2 Danish Center for Healthcare Improvements, Faculty of Social Sciences and Faculty of Health Sciences, Aalborg University, Aalborg East, Denmark

**Keywords:** Heart failure, Telemedicine, Randomized trial, Effectiveness, Cost-effectiveness, Denmark

## Abstract

**Background:**

Several heart failure studies have shown promising results for implementing telehealthcare. These studies have led to clinical and political interest in telehealthcare as a way to improve heart failure outcomes and lower costs. However, there is a need for large-scale clinical trials with cost-effectiveness assessments.

**Methods/design:**

The present study is known as the TeleCare North Heart Failure Trial in Denmark. We are studying the health effectiveness and cost-effectiveness of a telehealth (Telekit) solution compared with usual care for patients with heart failure. The design is a multicenter, two-arm, parallel-group, nonblinded, superiority randomized controlled trial. Outpatient healthcare centers will be responsible for recruiting eligible participants (600 participants are expected) for the trial in the geographic area of the North Denmark Region. Participants are qualified for inclusion if they have been diagnosed according to national guidelines and are categorized in New York Heart Association class 2, 3, or 4. Patients must have a permanent residence and be motivated to use telehealth care. The primary outcomes are changes in health-related quality of life (assessed using the Kansas City Cardiomyopathy Questionnaire, the EuroQol EQ-5D-5L questionnaire, and the Short Form Health Survey [SF-36]) and in the incremental cost-effectiveness ratio measured from baseline to follow-up. The secondary outcomes are changes in mortality and in physiological indicators such as blood pressure, pulse, and weight.

**Discussion:**

The TeleCare North Heart Failure Trial is intended to improve the international evidence base for the health effectiveness and cost-effectiveness of telehealthcare for patients with heart failure. The expectation is that the results of the trial can be generalized to all municipalities in Denmark and serve as an inspiration for further international research.

**Trial registration:**

ClinicalTrials.gov (NCT02860013). Registered on 28 July 2016.

**Electronic supplementary material:**

The online version of this article (doi:10.1186/s13063-016-1722-5) contains supplementary material, which is available to authorized users.

## Background

Heart failure (HF) is a condition in which the heart muscle is weakened and is therefore unable to pump blood sufficiently through the body [1, 2]. HF often develops after other conditions have damaged the heart. Some of the common causes of HF are coronary heart disease, diabetes, and high blood pressure. Approximately 26 million people worldwide are living with HF [3]. In many countries, population-based studies have found that approximately 1–2% of people have HF [4–6]. The prognosis for these patients is not good, with survival rates worse than those for many types of cancer. Moreover, HF induces great stress on patients, caregivers, and health systems. Demands on healthcare services are expected to increase greatly in the coming years as the number of patient diagnoses rise because of aging populations and damaging lifestyles. HF costs an estimated $20 billion each year in the United States [7]. This total cost includes the cost of medications to treat HF and the cost of healthcare services [1, 2].

Approximately 60,000–100,000 people in Denmark have HF, and the incidence of HF is increasing [8]. Moreover, there are approximately 11,000 hospitalizations per year for HF in Denmark [8]. The average age of patients with HF is 72 years, and the incidence increases with age. Approximately 5% of people over 75 have HF, and the proportion rises to over 10% for people over 85 years of age.

The prevention of HF-related disease and death needs to be made a worldwide healthcare priority [1, 2]. Despite the increasing number of people living with and dying as a result of HF, awareness of HF is low among the public, politicians, and some health professionals. Although there is no cure for HF, many cases are preventable, and most patients can be treated successfully to improve their quality of life and survival rate.

Studies of telehealthcare show positive results regarding health outcomes and quality of life in other patients with chronic illness [9]. Some studies of feasibility indicate that the use of telehealthcare may also lower the costs of healthcare [10–13]. Additionally, several studies have shown promising results for telehealthcare with regard to HF [14, 15], whereas others have not found an effect [16, 17]. These studies have led to increased political and clinical attention to telehealthcare as a means of improving outcomes for patients with HF and lowering costs. However, there is a need for large-scale clinical trials with cost-effectiveness assessments in telehealthcare research [14].

In Denmark, healthcare decision makers have agreed to support the advancement of research into the health effectiveness and cost-effectiveness of telehealthcare for patients with HF. As part of the national Danish plan for the dissemination of telemedicine, a full-scale randomized trial has been planned for the North Denmark Region (Region Nordjylland). The North Denmark Region is one of five regions in Denmark and has healthcare responsibility for approximately 580,000 people. The project is based on experiences with and solutions arising from the TeleCare North COPD trial [18]. The results of the trial will inform political and clinical decisions regarding how to implement a telehealthcare solution as a national standard for HF healthcare in Denmark.

The aims of this trial are separated into two focus areas. The first area aims to evaluate whether a specific telehealth solution that is serving as a supplement to usual healthcare increases quality of life for patients with HF compared with the quality of life of those receiving usual healthcare alone. The second area aims to evaluate the cost-effectiveness of this specific telehealth solution.

## Methods/design

### Study design

**Fig. 1:**
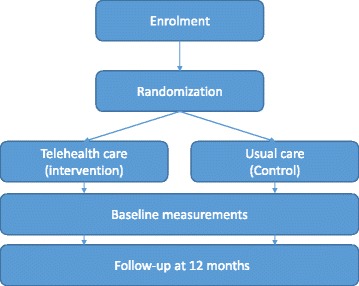
Flow diagram for inclusion, treatment, and follow-up

This trial, the TeleCare North Heart Failure Trial in Denmark, runs from 2016 to December 2017. It is a full-scale randomized trial with approximately 12 months of follow-up conducted throughout the whole North Denmark Region (Fig. 1). All results will be published in international journals at the end of the trial. (See Additional file 1 for information regarding the Standard Protocol Items: Recommendations for Interventional Trials [SPIRIT] checklist and Additional file 2 for the SPIRIT figure.)

### Participants

#### Inclusion criteria

All patients with HF who may benefit from telehealthcare are eligible for inclusion. Participants are qualified for inclusion if they have been diagnosed with HF according to national guidelines [19] and are categorized in New York Heart Association class 2, 3, or 4. Patients must have a permanent residence and be motivated to use telehealthcare. Motivation is simply assessed by the healthcare personnel by asking the patient prior to inclusion. Moreover, patients must have a landline or mobile phone, and they must be able to speak Danish or live with a relative who speaks Danish. In the latter case, the relative must be able to help the patient by translating information regarding the use of telehealthcare.

#### Exclusion criteria

Exclusion criteria are patients without a landline phone, a mobile phone, or GSM (global system for mobile communications) coverage; patients not able to understand Danish adequately to complete the questionnaires in the study. Comorbidity is not an exclusion criterion.

### Recruitment

Recruitment takes place in three outpatient healthcare centers in the North Denmark Region (Aalborg University Hospital, Vendsyssel Hospital, and Thy-Mors Hospital). The recruitment includes both newly diagnosed patients and people with an existing diagnosis of HF. For pragmatic reasons, recruitment will be consecutive; that is, the first patients recruited in the project will receive the intervention for a longer time span than those included later in the process. Clinic staff will be responsible for identifying potential participants and assessing eligibility when participants are in contact with the outpatient healthcare center for other reasons. The clinical staff are responsible for telling the patient about the trial, and a printed folder with information is handed to the patient. All relevant clinical staff have been informed in meetings by the trial administration and have been supplied with written information about how to identify potential participants and how to include them.

The outpatient healthcare centers will send signed informed consent forms, together with the patient questionnaires and baseline physical measurements, to the trial administrators. The patient questionnaires include questions about the participants’ baseline health-related quality of life (Kansas City Cardiomyopathy Questionnaire [KCCQ], EuroQol EQ-5D-5L questionnaire, and Short Form Health Survey [SF-36]) and demographic characteristics (age, sex, education level, comorbidities, smoking habits, marital status, and job status). The patient questionnaires are completed at the outpatient healthcare center before each patient is assigned to the telehealthcare (intervention) group or usual healthcare (control) group. If the patient feels more confident in answering the questionnaire at home, this will be possible. All primary outcome data will be entered electronically. Original study forms will be entered and kept on file.

### Randomization

The randomization of patients to either telehealthcare (intervention) or usual healthcare (control) is performed at the outpatient center after the patients have signed informed consent forms. Patient assignments are made using prepacked bundles of ten envelopes, each with a card, with assignment of five patients to the intervention group and five to the control group. Each prepacked bundle of ten envelopes is shuffled. In the outpatient healthcare center, a prepacked bundle is chosen to be the active bundle (i.e., the bundle used for assigning patients at random). When three envelopes are left in an active bundle, they are shuffled into a new bundle, and this new bundle is used as the active bundle.

### Intervention

#### Telekit

The telehealthcare solution, Telekit, is a tablet (Samsung Galaxy Tab 2; Samsung, Seoul, South Korea) that can be used to collect data from various external devices and disease-specific questionnaires. In the present study, the external devices will collect disease-specific data (blood pressure, pulse, and weight) from two devices and wirelessly transmit this data and the data from questionnaires to the central clinical system. The two external devices are a digital blood pressure monitor (Model UA-767 Plus BT-C; A&D Medical, Tokyo, Japan) and a scale (Precision Health Scale, UC-321PBT-C; A&D Medical). The software for the tablet, including the framework for the disease-specific questionnaires, was developed by Silverbullet (Aarhus, Denmark; www.opentele.silverbullet.dk). The questionnaires were constructed by clinical domain experts in the North Denmark Region. The tablet actively reminds the patients when it is time to take measurements and answer questionnaires. The patients, their relatives, and general practitioners can access the collected data via a public website (https://www.sundhed.dk/).

#### Starting the intervention

After randomization, patients with HF in the intervention group will be contacted (via telephone) by their municipality. The Telekit (including external devices) is ordered through Atea (Atea Danmark, Aalborg, Denmark), which delivers and installs the Telekit at home with the citizen. A nurse from the HF patient’s municipality will instruct the patient in the use of the Telekit. The municipality nurse will show the patient how to use the tablet and how to take physical measurements and will guide the patient through the Telekit manual before letting the patient try the Telekit him- or herself. Patients will be asked to use the scale and blood pressure monitor daily during the first 2 weeks and one to two times weekly after the first 2 weeks. A follow-up visit is scheduled between 2 and 4 weeks after the first appointment to ensure that the patient uses the Telekit properly.

### Measurement assessment

The data, which include measurements (blood pressure, pulse, and weight) and symptom-specific information from the tablet questionnaire will be assessed by a specially trained nurse. The responsibility is shared between the municipalities and the outpatient healthcare centers, depending on where the patients are affiliated.

Data assessment implies that the patient’s data are seen, interpreted, and evaluated to determine whether there should be an intervention. The data assessments will be conducted at fixed intervals (typically one or two times per week). After each assessment, an acknowledgement will be sent to the patient. If a patient’s measurements are not received as expected, the patient will be contacted. The measurements are classified as being within the normal range or outside the normal range. These predefined thresholds are adjusted if necessary during the first 14 days of measurements to fit the individual patient. The predefined thresholds are systolic blood pressure 170–100 mmHg, diastolic blood pressure 90–50 mmHg, pulse 80–55 beats/minute, and weight ±2 kg.

If the clinician observes abnormal measurements, several options are available:

1. The clinician can contact the patient and ask for a new measurement if the measurement is considered to be less suitable.
2. The clinician can contact the patient to assess the patients condition.
3. It is possible to start a self-treatment plan for the patient.
4. If the clinician judges that the patient is cable of taking contact to his or her own general practitioner, the patient is asked to do so.
5. The clinician can contact the patient’s general practitioner directly.

The intervention needed is always a clinical judgment based on the patient’s measurement and possibly a subsequently contact with the patient. It should be noted that the goal is to help the patient understand his or her own data and thereby empower the patient to take responsibility for handling the disease.

### Outcome assessments

#### Effectiveness

All outcome measures for the two groups (usual care group and the intervention group) are collected at baseline and follow-up. The primary outcome is change in health-related quality of life (SF-36 and KCCQ) from baseline to follow-up. SF-36 total score is used as the primary outcome, and the subscales are used as secondary outcomes. The secondary outcomes for effectiveness are changes in the mortality rate and physiological measurements such as blood pressure, pulse, and weight from baseline to follow-up. Mortality rate is considered only if enough participants are included to satisfy the power calculation for this secondary outcome. The clinical impact of telehealthcare is considered to be positive if no increase in mortality is observed and if pulse and blood pressure are lowered.

#### Cost assessment

The main outcome for cost is the incremental cost-effectiveness ratio (ICER). The ICER is measured as the total cost per quality-adjusted life-year (QALY) gained from baseline to follow-up. It is anticipated that telehealthcare will increase the participants’ quality of life, but no difference in mortality is expected. Additionally, it is expected that telehealthcare will reduce cost, particularly due to a reduction in the number of admissions/readmissions and outpatient visits [20].

### Sample size

A sample size calculation for effectiveness was performed using OpenEpi (http://www.openepi.com/Menu/OE_Menu.htm). Conservative estimates were applied for the minimal clinically important difference (change equal to 5 for the SF-36 physical component summary). With a power of 80%, groups of equal size, a standard deviation of 15, and a two-sided *p* value of <0.05, the required sample size is estimated to be at least 284 participants. With an expected loss to follow-up of 10%, the total required sample size is estimated to be at least 316 participants.

### Statistical methods

Statistical analysis will be conducted using an intention-to-treat standard. The results will be presented according to the Consolidated Standards of Reporting Trials (CONSORT) statement for randomized clinical trials [21]. Missing data will be assessed to identify patterns of missing data. Multiple imputation will be used if necessary, and the observations will be described according to epidemiological guidelines [22]. If appropriate, an adjustment will be made for differences between the intervention group and the control group with regard to baseline characteristics.

#### Effectiveness

To evaluate effectiveness, differences between the intervention group and the control group with regard to SF-36 scores, mortality rates, and physical measurements from baseline to follow-up will be analyzed. The outcome will primarily be analyzed using two-sample paired statistics (e.g., two-sample paired *t* test), such that the differences from baseline to follow-up will be assessed between groups.

#### Cost assessment

To evaluate economic consequences, a health economic evaluation will be conducted. This will include the estimation of an ICER to summarize the cost-effectiveness of the telehealthcare intervention compared with the control group treatment. The ICER is defined as the difference in cost between two possible interventions, divided by the difference in their effect. The ratio represents the average incremental cost associated with one additional unit of the measure of effect [22].

The effect measure used for the health economic evaluation will be QALYs. For all patients, the effect will be estimated using the EQ-5D-5L questionnaire and the associated Danish societal weights [23].

A health sector perspective will be used to estimate the incremental costs. This means that for all patients, all costs related to healthcare services should be included. This includes inpatient and outpatient hospital services, general practitioner services, home care, and prescription medicine. Costs that are associated only with the research project that would not occur in a real-life setting will be excluded. Costs associated with the telehealthcare intervention will be calculated and included in the ICER.

Denmark is internationally known for having a comprehensive registration of data on healthcare activities. All citizens have a social security number (CPR) that is registered when in contact with a public service, such as for healthcare services. Therefore, data may be extracted for individuals to obtain a fairly accurate estimate of that individual’s use/cost of healthcare services and/or resources across municipalities, general practitioners, and hospitals.

Both deterministic and probabilistic sensitivity analyses will be conducted to assess the uncertainty of the results. A Bayesian approach will be applied using bootstrapping techniques to estimate credibility intervals for the costs and outcomes. ICERs will be estimated using second-order Monte Carlo simulations and presented in a scatterplot and a cost-acceptability curve [23].

### Ethics

The trial has been authorized by the Danish Data Protection Agency. The study is being conducted in accordance with the Helsinki declaration. The trial has been presented to the Ethical Committee for Medical Research in the North Denmark Region; this committee decided that no ethical approval was necessary. All study-related information will be stored securely at the study site.

## Discussion

There is a need for large-scale clinical trials that include cost-effectiveness assessments in telehealthcare research. This study, the TeleCare North Heart Failure Trial, is intended to improve the international evidence base regarding the health effectiveness and cost-effectiveness of telehealthcare for patients with HF. The TeleCare North Heart Failure Trial is a large-scale, pragmatic randomized clinical trial that recruits participants from the entire geographic area of the North Denmark Region. Changes in health-related quality of life and health costs are evaluated.

It is expected that the results of the TeleCare North Heart Failure Trial could be generalized to all regions in Denmark. Decision makers have indicated that if the telehealthcare solution in this trial proves to be cost-effective for patients with HF, it could serve as a national standard for telehealthcare for patients with HF.

## Trial status

Recruitment is ongoing.

## Additional files

Additional file 1: SPIRIT 2013 checklist: recommended items to address in a clinical trial protocol and related documents. (DOC 119 kb)
Additional file 2: Schedule of enrollment, interventions, and assessments. (DOC 46 kb)

## Abbreviations

CONSORT: Consolidated Standards of Reporting Trials
GSM: Global system for mobile communications
HF: Heart failure
ICER: Incremental cost-effectiveness ratio
KCCQ: Kansas City Cardiomyopathy Questionnaire
SF-36: Short Form Health Survey
QALY: Quality-adjusted life-year
SPIRIT: Standard Protocol Items: Recommendations for Interventional Trials

## References

[1] Ponikowski P, Anker SD, Al-Habib KF, Cowie MR, Force TL, Hu S (2014). Heart failure: preventing disease and death worldwide.

[2] Krum H, Abraham WT (2009). Heart failure. Lancet.

[3] Bui AL, Horwish TB, Fonarow GC (2011). Epidemiology and risk profile of heart failure. Nat Rev Cardiol.

[4] Blair JE, Huffman M, Shah SJ (2013). Heart failure in North America. Curr Cardiol Rev.

[5] Guo Y, Lip GY, Banerjee A (2013). Heart failure in East Asia. Curr Cardiol Rev.

[6] Al-Shamiri M (2013). Heart failure in the Middle East. Curr Cardiol Rev.

[7] Heidenreich PA, Trogdon JG, Khavjou OA, Butler J, Dracup K, Ezekowitz MD (2011). Forecasting the future of cardiovascular disease in the United States: a policy statement from the American Heart Association. Circulation.

[8] Hjertesvigt KJ. Sundhed.dk; 2015. https://www.sundhed.dk/sundhedsfaglig/laegehaandbogen/hjerte-kar/tilstande-ogsygdomme/hjertesvigt/hjertesvigt/.

[9] Polisena J, Tran K, Cimon K, Hutton B, McGill S, Palmer K (2010). Home telehealth for chronic obstructive pulmonary disease: a systematic review and meta-analysis. J Telemed Telecare.

[10] de Toledo P, Jiménez S, del Pozo F, Roca J, Alonso A, Hernandez C (2006). Telemedicine experience for chronic care in COPD. IEEE Trans Inf Technol Biomed.

[11] Haesum LKE, Soerensen N, Dinesen B, Nielsen C, Grann O, Hejlesen O (2012). Cost-utility analysis of a telerehabilitation program: a case study of COPD patients. Telemed J E Health.

[12] Johnston B, Wheeler L, Deuser J, Sousa KH (2000). Outcomes of the Kaiser Permanente Tele-Home Health Research Project. Arch Fam Med.

[13] Paré G, Poba-Nzaou P, Sicotte C, Beaupré A, Lefrançois É, Nault D (2013). Comparing the costs of home telemonitoring and usual care of chronic obstructive pulmonary disease patients: a randomized controlled trial. Eur Res Telemed.

[14] Louise AA, Tuner T, Gretton M, Baksh A, Cleland JG (2003). A systematic review of telemonitoring for the management of heart failure. Eur J Heart Fail.

[15] Anker SD, Koehler F, Abraham WT (2011). Telemedicine and remote management of patients with heart failure. Lancet.

[16] Chaudhry SI, Mattera JA, Curtis JP, Spertua JA, Herrin J, Lin Z (2010). Telemonitoring in patients with heart failure. N Engl J Med.

[17] Koehler F, Winkler S, Schieber M, Sechtem U, Stangl K, Böhm M (2011). Impact of remote telemedical management on mortality and hospitalizations in ambulatory patients with chronic heart failure: the Telemedical Interventional Monitoring in Heart Failure Study. Circulation.

[18] Udsen FW, Lilholt PH, Hejlesen O, Ehlers LH (2014). Effectiveness and cost-effectiveness of telehealthcare for chronic obstructive pulmonary disease: study protocol for a cluster randomized controlled trial. Trials.

[19] Dansk Cardiologisk Selskab. Nationale behandlingsvejledning 2015. Dansk Cardiologisk Selskab; 2015.

[20] Dinesen B, Haesum LKE, Soerensen N, Nielsen C, Grann O, Hejlesen O (2012). Using preventive home monitoring to reduce hospital admission rates and reduce costs: a case study of telehealth among chronic obstructive pulmonary disease patients. J Telemed Telecare.

[21] Campbell MK, Piaggio G, Elbourne DR, Altman DG (2012). Consort 2010 statement: extension to cluster randomised trials. BMJ.

[22] Sterne JC, White IR, Carlin JB, Spratt M, Royston P, Kenward MG (2009). Multiple imputation for missing data in epidemiological and clinical research: potential and pitfalls. BMJ.

[23] Wittrup-Jensen K (2009). Generation of a Danish TTO value set for EQ-5D health states. Scand J Public Health.

