# Supplementary material for: Health effectiveness and cost-effectiveness of telehealthcare for heart failure: study protocol for a randomized controlled trial
Source: Trials. 2016 Dec 12;17:590. doi: 10.1186/s13063-016-1722-5 (PMC5154081; doi:10.1186/s13063-016-1722-5)
Supplement: Additional file 2: — Schedule of enrollment, interventions, and assessments. (DOC 46 kb) [file 13063_2016_1722_MOESM2_ESM.doc]

Figure 2 Schedule of enrolment, interventions, and assessments.

|  | **STUDY PERIOD** | | | | | | | |
| --- | --- | --- | --- | --- | --- | --- | --- | --- |
|  | **Enrolment** | **Allocation** | **Post-allocation** | | | | | **Close-out** |
| **TIMEPOINT** | ***-t_1_*** | **0** | ***12-month*** | | | | | ***t_x_*** |
| **ENROLMENT:** |  |  |  |  |  |  |  |  |
| **Eligibility screen** | X |  |  |  |  |  |  |  |
| **Informed consent** | X |  |  |  |  |  |  |  |
| **Allocation** |  | X |  |  |  |  |  |  |
| **INTERVENTIONS:** |  |  |  |  |  |  |  |  |
| ***Telehealth care*** |  |  |  |  |  |  |  |  |
| ***Usual Care*** |  |  |  |  |  |  |  |  |
| **ASSESSMENTS:** |  |  |  |  |  |  |  |  |
| ***SF 36*** | X |  |  |  |  |  |  | X |
| ***EQ-5D-5L*** | X |  |  |  |  |  |  | X |
| ***KCCQ*** | X |  |  |  |  |  |  | X |
